# Spontaneous biliary tract perforations: an unusual cause of peritonitis in pregnancy. Report of two cases and review of literature

**DOI:** 10.1186/1749-7922-1-21

**Published:** 2006-07-10

**Authors:** Nikhil Talwar, Manoj Andley, Bina Ravi, Ajay Kumar

**Affiliations:** 1 Department of Surgery, Lady Hardinge Medical College, University of Delhi, New Delhi-110001, India

## Abstract

Spontaneous perforations of the biliary tract are rare in adults and even more so during pregnancy. Perforation of the gall bladder is a potentially fatal complication of cholecystitis. The infrequency of perforation in the setting of calculous disease of the gall bladder is probably due to the thickened wall of the organ that has long been the seat of chronic inflammation. Common bile duct perforations have been reported in adults most commonly in association with choledocholithiasis. The diagnosis of biliary tract perforations is often delayed due to their non specific symptoms, which results in high morbidity. Early diagnosis and aggressive therapy are mandatory to alleviate this condition. Delayed diagnoses and treatment may have more serious consequences for pregnant women than for other patients. Very few cases of biliary tract perforations have been reported in pregnant women. We report two such cases in pregnancy: first of a gall bladder perforation associated with cholelithiasis and the second of a common bile duct perforation in pregnancy in which no apparent cause was found.

## Background

The incidence of biliary tract disease during pregnancy ranges from 0.05% to 0.3% [1]. Despite the rarity of the condition, complications of gallstones represent the second most common nongynecologic condition requiring surgery in pregnancy after appendicitis [2]. The most common indications for intervention for gall stones during pregnancy include: obstructive jaundice, acute cholecystitis failing medical management, and gall stone induced pancreatitis [3]. Gall bladder perforation has been reported to occur in 3 to 10% cases of acute cholecystitis in adults; however, it has rarely been reported in pregnancy [4]. Risk factors for gall bladder perforation in adults include: age greater than 60 years, immunosupression, steroid use, and severe systemic disease [4]. Since this condition is unusual during pregnancy, accurate diagnosis and treatment may be delayed resulting in perinatal morbidity.

Spontaneous perforation of the common bile duct is a rare event in adults, with only 40 cases having been reported earlier [4]. Cholelithiasis, choledocholithiasis, and tumor obstruction of the ampulla have been reported as possible etiologies of perforation [5]. We report two cases of spontaneous biliary tract perforations in pregnancy, which were managed successfully. The clinical presentations, radiological findings and management are discussed.

## Clinical presentation

### Case 1

A 28-year-old gravida 2, para 1, 30 weeks pregnant woman presented with sudden onset abdominal pain and distension for two days. There was previous history of right upper abdominal pain for six months, which got relieved on analgesics. The previous pregnancy had been uneventful. On physical examination, she was moderately dehydrated and her temperature was 39.6°C. Her pulse was 110/minute, blood pressure was 90/60 mmHg and there was no pallor or icterus. On abdominal examination, there was generalized tenderness, guarding, rigidity and rebound tenderness. Shifting dullness and fluid thrill was present. The uterine fundal height corresponded to 30 weeks gestation. Bowel sounds were absent. Routine laboratory tests revealed hemoglobin of 10.4 g/dL, total leukocyte count of 18850 cells per mL. The blood sugar, kidney and liver function tests were normal. The chest X ray was normal and there was no free air under the diaphragm. Abdominal sonography revealed bowel loops floating in large amount of free fluid. A Single live foetus of 30 weeks gestation was seen. Gall bladder with multiple calculi and wall thickness of 4 mm was also noted. Paracentesis yielded a free flow of purulent fluid admixed with bile.

A clinical diagnosis of peritonitis due to bowel perforation was made and the patient underwent an exploratory laparotomy, which revealed about 4 litres of purulent fluid inside the peritoneal cavity. A thorough exploration revealed normal bowel. A gall bladder with multiple stones and a perforation at the fundus was seen. A cholecystectomy with peritoneal lavage was performed. Postoperative course was uneventful and the patient was discharged one week later. Histopathology of the gall bladder was suggestive of acute cholecystitis. Her pregnancy continued and at 37 weeks' gestation she vaginally delivered a healthy baby. The patient is asymptomatic after a follow up of three years.

### Case 2

**Figure 1:**
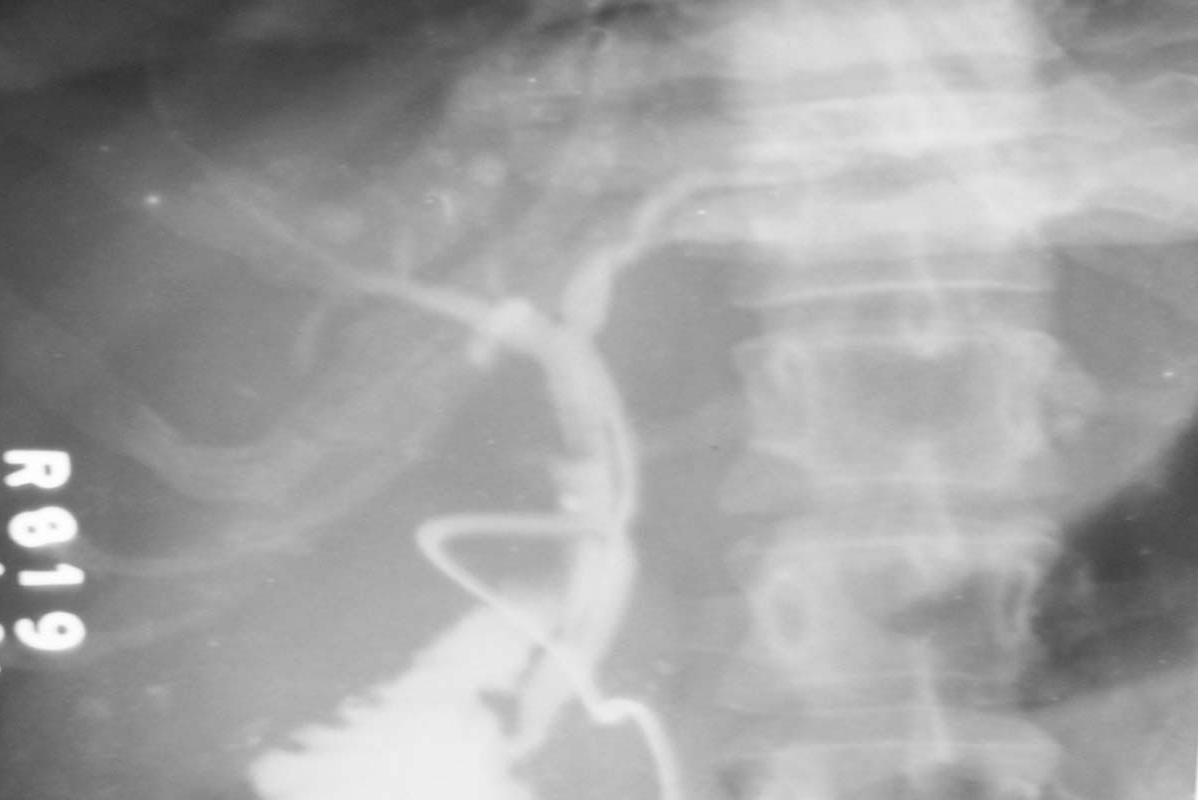
Postoperative T-tube cholangiogram of case 2 showing normal flow of the contrast into the biliary radicals and the duodenum.

A 21-year-old primipara with 32 weeks pregnancy was referred to us from the gynecology department with upper abdominal pain and high-grade fever for 3 days associated with abdominal distension. On examination, the patient had a pulse of 124/minute and her blood pressure was 96/64 mmHg. Mild pallor was noted and there was no icterus, lymphadenopathy or pedal edema. On abdominal examination, the patient had generalized tenderness, guarding, rigidity and rebound tenderness. Bowel sounds were absent. The uterine fundal height corresponded to 32 weeks' gestation. Routine laboratory tests revealed hemoglobin of 12.1 g/dL, total leukocyte count of 14100 cells per mL. The blood sugar, kidney and liver function tests were normal. Abdominal sonography showed a viable fetus of 32 weeks gestation. There was a large collection of fluid in the right side of peritoneal cavity below the liver. Other viscera were normal. Ultrasound guided diagnostic aspiration of the collection revealed frank bile, the presence of which confirmed by biochemical analysis of the fluid. With the presumptive diagnosis of duodenal ulcer perforation peritonitis, the patient was taken up for an exploratory laparotomy. There was an 800 mL collection of bile in the subhepatic and right paracolic space. The stomach and intestines were normal. The gall bladder was distended and a 0.5 × 0.5 cm perforation in the lateral wall of the supraduodenal portion of the CBD was seen. There was no evidence of gall stones. The CBD perforation was closed over a T-tube. The midline incision was closed after inserting a drain in the subhepatic space. The patient went into spontaneous labor 48 hours later and a preterm baby was delivered. Subsequently, on 15^th ^postoperative day, after a normal T-tube cholangiogram (figure 1), the T-tube was removed and the patient was discharged the next day. The mother and child are doing well after a follow up period of one year.

## Conclusion

The most common non-obstetric cause of peritonitis in pregnancy is appendicitis. Biliary tract perforations are unusual causes of peritonitis in pregnancy. The signs and symptoms are often nondiagnostic, especially during pregnancy, and diagnosis may be delayed with possible fatal consequences [4]. Gall bladder or CBD perforations as a cause of peritonitis in pregnancy have been rarely reported in literature and their exact incidence in pregnancy is not known. Even when the gall bladder perforates, the usual outcome is a local abscess, on account of the adhesion that form between the gall bladder, greater omentum and the parietal peritoneum [6]. Although gall bladder perforation has been reported to occur in 3 to 10% cases of acute cholecystitis in adults, gall bladder perforation into the general peritoneal cavity is even rare, occurring in only 0.5% of the patients undergoing conservative management for acute cholecystitis. The initiating event in majority of these patients is impaction of the stone leading to epithelial injury and ischemia due to distension of the gall bladder. The site of perforation is either at the fundus, which is farthest away from the blood supply, or less commonly at the neck from the pressure of an impacted stone [7]. Abdominal Paracentesis is helpful in diagnosis of biliary peritonitis. In the presented case, abdominal paracentesis lead to a timely diagnosis of biliary peritonitis and patients were operated without delay. But gall bladder perforation was not suspected preoperatively. The surgical management consists of cholecystectomy, copious irrigation and drainage of the abdominal cavity [4].

Only about 40 cases of spontaneous rupture of the CBD have been reported earlier and it is extremely rare in pregnancy [8]. It may result from increases intraductal pressure due to stones, thrombosis of a mural vessel, intraluminal infection in the bile duct wall, infected diverticulum of the duct or reflux of pancreatic secretions [5]. In the present case of CBD perforation, no gall stones were found. Recommended treatment includes cholecystectomy and CBD exploration with T-tube drainage in cases of small perforations. Roux-en-Y biliary-enteric anastomosis is indicated if the ductal disruption is large [8].

A high index of suspicion and an early surgical intervention are the mainstays of therapy of peritonitis in pregnancy and may be associated with decreased maternal and fetal morbidity. In a pregnant lady with peritonitis, if a biliary tract perforation is detected intra-operatively, the treatment should be based on the conventional surgical principles of treating such conditions.

## Abbreviations

1. CBD common bile duct.

2. g/dL grams per deciliter.

3. mL milliliter.

4. % percent.

5. cm centimeter.
